# Investigation the global effect of rare earth gadolinium on the budding *Saccharomyces cerevisiae* by genome-scale screening

**DOI:** 10.3389/fmicb.2022.1022054

**Published:** 2022-11-28

**Authors:** Yuhang Cao, Caiyun Zhang, Yu Fang, Yumeng Liu, Kexin Lyu, Jian Ding, Xue Wang

**Affiliations:** School of Life Sciences and Medicine, Shandong University of Technology, Zibo, China

**Keywords:** global effect, gadolinium, sensitive genes, genome-scale screening, yeast

## Abstract

**Introduction:**

The rare earth gadolinium (Gd) is widely used in industry and medicine, which has been treated as an emerging pollutant in environment. The increasing pollution of Gd has potential hazards to living organisms. Thus it is essential to investigate the toxicity and action mechanism of Gd in biological system.

**Methods:**

In this study, the global effect and activation mechanism of Gd on yeast were investigated by genome-scale screening.

**Results and discussion:**

Our results show that 45 gene deletion strains are sensitive to Gd and 10 gene deletion strains are Gd resistant from the diploid gene deletion strain library of *Saccharomyces cerevisiae*. The result of localization analysis shows that most of these genes are involved in cell metabolism, cell cycle, transcription, translation, protein synthesis, protein folding, and cell transport. The result of functional analysis shows that four genes (*CNB1, CRZ1, VCX1,* and *GDT1*) are involved in the calcium signaling pathway, and four genes (*PHO84, PHO86, PHO2,* and *PHO4*) are involved in phosphorus metabolism. For Gd^3+^ has the similar ion radius with Ca^2+^ and easily binds to the phosphate radical, it affects Ca^2+^ signaling pathway and phosphorus metabolism. The genes *ARF1, ARL1, ARL3, SYS1, COG5, COG6, YPT6, VPS9, SSO2, MRL1, AKL1,* and *TRS85* participate in vesicle transport and protein sorting. Thus, Gd accumulation affects the function of proteins related to vesicle transport, which may result in the failure of Gd transport out of cells. In addition, the intracellular Gd content in the 45 sensitive deletion strains is higher than that in the wild type yeast under Gd stress. It suggests that the sensitivity of yeast deletion strains is related to the excessive intracellular Gd accumulation.

## Introduction

Rare earth elements (REEs) include 17 chemical elements (lanthanum-luteum, scandium, and yttrium) (Gwenzi et al., 2018). Depending on the physical and chemical properties, REEs can be classified as light and heavy rare earths. Gadolinium (Gd) is one of heavy rare earth elements, and it is mostly present in the form of compounds. The concentration of Gd in the soil environment is 1.6 ~ 7.1 mg/kg in China, and it is 0.2 ~ 36 mg/kg worldwide (Ramos et al., 2016). The concentration of Gd is 0.36 ~ 26.9 ng/l in marine systems, 0.347 ~ 80 μg/l in freshwater environments, and it reached 409.4 ng/l in a submarine outfall (Trapasso et al., 2021).

At present, Gd has been widely used in magnetic refrigeration, medical treatment, and nuclear energy (Gwenzi et al., 2018; Ben Salem and Barrat, 2021; Itoh et al., 2021). Therefore, the wide application of Gd has made it become a new emerging contaminant of aquatic environment (Rogowska et al., 2018). In the medical field, Gd contrast is widely used in magnetic resonance imaging (MRI) to improve the imaging signal and enhance magnetic resonance angiography (MRA) (Kim et al., 2018; Yon et al., 2019; Sakol et al., 2020). However, it is also observed that Gd-containing contrast has side effects to a small number of people (Raczeck et al., 2019). In addition, the toxicity of Gd on rice seedlings shows that the higher concentration of Gd inhibits the growth of rice and Gd can be accumulated in rice (Zhang et al., 2019a).

Microorganisms are widely used for preventing heavy metal pollution. Various biosorption materials, such as yeasts, bacteria and algae, have been used to remove metal ions in water environment. Biosorption is an ideal biological materials for treating wastewaters contaminated by heavy metals (Veglio and Beolchini, 1997; Gaur et al., 2014; Razzak et al., 2022). In the beverage and food industry, yeast *Saccharomyces cerevisiae* is easily to cultivate at a large scale and possesses various advantages as a metal biosorbent (Kapoor and Viraraghavan, 1995; Sagar Jena et al., 2022). Moreover, *S. cerevisiae* is a simple, easily operable and flexible unicellular eukaryotic organism, and it is one of the most widely used eukaryotic model organisms in laboratory (Orr-Weaver et al., 1983; Baudin et al., 1993; Wach et al., 1994; Nelissen et al., 1997). As a eukaryotic model organism, *S. cerevisiae* is extensively used to investigate the mechanism of metal ion transport (Eide, 2000; Revel et al., 2022).

In addition, the genome-wide information of yeast has been acquired with the completion of genome sequencing. Compared to the other model organisms, yeast reproduces quickly and can reproduce 1 generation in 1.5 ~ 2 h (Baudin et al., 1993). In addition, the growth of yeast can be divided into haploid and diploid, which is the advantage of yeast in the studies on gene functions. The genome of *S. cerevisiae* is simpler compared to *Caenorhabditis elegans* and human. The apart between two intercoding protein genes are approximately 6 kb in *C. elegans*, and it is at least 30 kb in human, while it is only 2 kb in yeast (Baudin et al., 1993).

It is known that some metal ions are essential nutritional elements for organisms. However, the over-accumulation of metal ions is toxic for organisms. Fortunately, the cellular homeostasis mechanism and the detoxification system precisely control the level and distribution of metal ions in cells. At present, the functional genomics is rapidly accelerating research on the metal ion stresses in yeast (Eide, 2001; Shao et al., 2018; Akao, 2019). If yeast cells are exposed to the extracellular environment stress, various signal transduction pathways will be stimulated in the response to stress. Previously, Du et al. found that *PHO4* gene encodes a transcription factor of the myc family helix–loop–helix (bHLH) structure, and it is activated under low phosphate stress (Du et al., 2015). In addition, Yoshimoto et al. observed that the Ca^2+^/calcineurin signaling pathway was activated by various stress factors, such as exposure to high Ca^2+^ and Na^+^ stresses (Yoshimoto et al., 2002). Cunningham et al. found that *PMC1* (Ca^2+^-ATPases on the plasma membrane) plays a key role in calcineurin activation through regulating Ca^2+^ concentration in yeast (Cunningham and Fink, 1994).

As a fully sequenced eukaryotic cell, yeast *S. cerevisiae* is one of the main experimental models for understanding eukaryotic systems, especially for studying the mechanism of metal ion toxicity (Horstmann and Kim, 2021; Robinson et al., 2021). At present, *S. cerevisiae* has been widely used as a model for studying metal ion stresses and related signal transduction pathway. Previously, various signaling pathways and ion transporters has been found in yeast under metal stresses (Cools et al., 2019; Johnston and Strobel, 2019; Zhang et al., 2019b; Kumari et al., 2021; Shi et al., 2021). Rare earth element Gd is a new emerging contaminant of environment, and Gd^3+^ has the similar ion radius with Ca^2+^ and easily binds to the phosphate radical. To fully understand the global effect and regulatory mechanism of Gd in eukaryotic cells, we screened the diploid gene deletion library of yeast under Gd stress. And, we identified 45 Gd sensitive deletion strains and 10 resistance gene deletion strains in total. The functional localization analysis was further performed to investigate the mechanism of Gd detoxification and transportation in yeast.

## Materials and methods

### Yeast strains and culture

The diploid *S. cerevisiae* strains (BY4743 genetic background and KanMX4 genetic labels with G418 resistant) were purchased from Invitrogen Inc. (USA). The yeast deletion library BY4743 was created by a collaboration of eight North American and eight European laboratories composing the Saccharomyces Genome Deletion (SGD) Project. The intent of this consortium was to produce a deletion clone for each gene in the yeast genome. The library consists of 4,741 homozygous diploid clones. Yeast was grown at 30°C in the YPD medium (2% peptone, 1% yeast extract, and 2% glucose, pH 5.6).

### Screening Gd-sensitive and Gd-resistant phenotype of yeast

To find a sublethal concentration of Gd to *S. cerevisiae*, we firstly designed the effect of 0, 1, 2, 3, and 4 mM Gd on the yeast growth, and we observed that 4 mM Gd was lethal for yeast. For the status of yeast growth was good at 3 mM Gd and 4 mM Gd was lethal for yeast, we further designed three additional Gd concentrations (3, 3.5, and 4 mM Gd) to study the effect of Gd on yeast growth. The detailed method was as follows. The experiment for screening Gd-sensitive and Gd-resistant phenotype of yeast was repeated three times.

Firstly, the single colonies of yeast were incubated in 3 ml YPD liquid medium and shaken at 200 rpm for 16 h at 30°C. The yeast solution was diluted and transferred to YPD medium containing 0, 1, 2, 3, and 4 mM Gd(NO_3_)_3_ (Aladdin Co., Ltd., Shanghai, China). The size of bacterial plaque was observed after 2 days incubation at 30°C to investigate the sublethal concentration of Gd to *S. cerevisiae*.

Then, according to the protocal of a previous study (Luo et al., 2016), 0, 3, 3.5, and 4 mM Gd(NO_3_)_3_ was further used for Gd toxicity preliminary screening. We used the library strains for the preliminary screening of fungi mutations that were sensitive to Gd. All strains were copied to the YPD medium containing 0 or 3.5 mM Gd by using 384 pin tool, respectively. Two days later, the tablet was photographed and analyzed the growth of each mutant. Based on the analysis of colony size, 3.5 mM Gd nitrate was used for screening Gd-sensitive mutants. Compared to the surrounding mutants, if the colony size of mutant was reduced more than 30% by Gd treatment, it was considered Gd-sensitive.

Finally, we performed the re-screening and verification of Gd-sensitive mutants by the method of continuous yeast culture dilution determination (Jiang et al., 2014). The sensitive mutants were streak cultured from the original library to the YPD liquid medium, and inoculated into YPD liquid medium for 12 h. The corresponding yeast strains from preliminary screening were cultured and purified on YPD solid plates for 48 h at 30°C to acquire the single colonies of yeast. The single colonies were inoculated into YPD liquid medium and cultured for 16 h at 30°C in an incubator with 200 rpm. After adjusting the concentration of bacteria solution, we obtained five yeast culture diluents (10^−0^, 10^−1^, 10^−2^, 10^−3^, 10^−4^). Then, five yeast culture diluents (10^−0^, 10^−1^, 10^−2^, 10^−3^, 10^−4^) were put on the YPD solid medium containing 0, 3.5 mM Gd(NO_3_)_3_, and 10.5 mM NaNO_3_ (Aladdin Co., Ltd., Shanghai, China), respectively. To confirm that the mutant was sensitive to Gd^3+^ rather than NO_3_^−^, 10.5 mM NaNO_3_ was used for the wild-type comparison (Luo et al., 2016). The yeasts were cultured at 30°C for 2 ~ 5 days and analyzed the growth and plaque size of each mutant. In addition, for screening Gd-resistant phenotype of yeast, 0, 3.8 mM Gd(NO_3_)_3_, and 10.5 mM NaNO_3_ were used according to the above method.

### Detecting the intracellular Gd concentration

The intracellular Gd concentration was detected according to a previous method. (Zhao et al., 2013). For 3.5 mM Gd was lethal for various yeast strains, we used 1.75 mM Gd to detect the intracellular Gd concentration. The yeast strains were seeded into 5 ml sterilized medium and incubated at 30°C with 220 rpm shaking for 16 h. Then appropriate volume of bacterial solution was transferred to 50 ml medium containing 0 and 1.75 mM Gd, and cultured at 30°C with 220 rpm shaking for 4 h. The yeast were collected by centrifugation at 2000 rpm for 2 min at 4°C. Then, the yeasts were washed with 10 mM MgCl_2_ (containing 1 M sorbitol), and centrifuged at 2000 rpm for 2 min at 4°C. The yeasts were lysed in 10 ml 10 mM MgCl_2_. 0.3 ml bacterial solution was diluted 10 times with MgCl_2_, and the OD value was measured at 600 nm to adjust bacterial solution for consistent concentrations. Finally, the remaining 2.7 ml solution was added into 50 μl 6 M HNO_3_ and nitrated at 95°C for 60 min. After centrifugation at 2000 rpm for 2 min at 4°C, the supernatant was collected to analyze the intracellular Gd concentration with an inductively coupled plasma mass spectrometer (ICP-MS, Agilent 7,500). Six single mutant colonies were detected and the wild type BY4743 was used as control. The experiment was repeated six times.

### Testing the missing strain genome by PCR

To test the missing strain genome, each genome of yeast missing strain was used as a template for PCR. Its upstream primer is designed within 100 ~ 300 bp upstream of the mutant gene open reading box, and the downstream primer is designed at the internal sequence of the KanMX4 knockout box (about 1,200 bp from initiation codon). The primers used for testing are listed in Supplementary Table 1. The experiment was repeated three times.

### Gene function and localization analysis

The localization and function of the corresponding genes of Gd-sensitive mutants were annotated by using MIPS, the yeast genome database,^1^ FunSpec,^2^ and BioGRID.^3^ In addition, the protein interactome (protein–protein interactions) was further analyzed in yeast. The proteins corresponding to Gd sensitive gene deletion strains were mapped onto the interactome, and then filtered to identify the connected groups of proteins.^4^ The minimum required interaction score was set at high confidence 0.7.

### Data analysis

Data was expressed as mean ± SEM. The difference among various experimental groups was analyzed with the single factor variance analysis (LSD’s test) analysis by using the statistical software SPSS (16.0). The level of significance was set at *p* < 0.05.

## Results

### Genes involved in the Gd sensitivity of yeast cells

**Figure 1:**
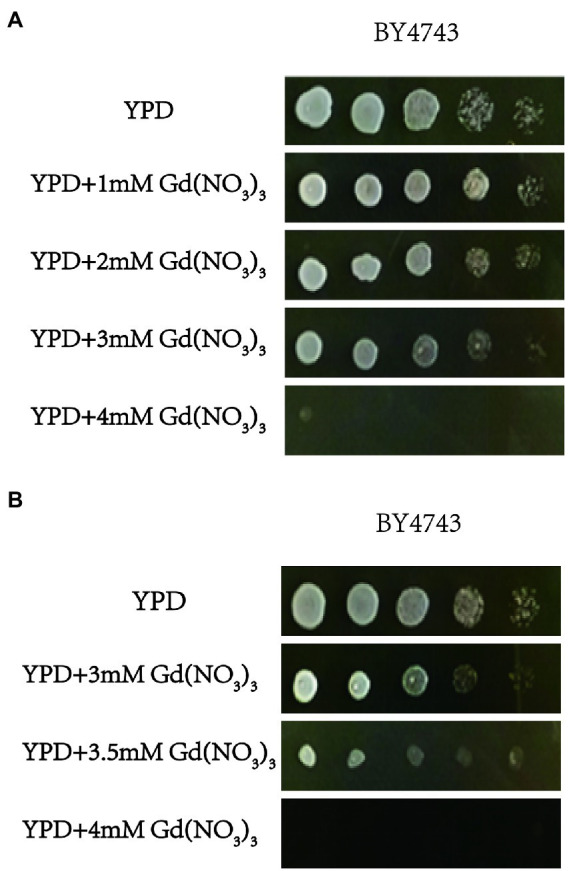
The toxicity of Gd and screening Gd-sensitive concentration. **(A)** Effect of 0, 1, 2, 3, and 4 mM Gd on yeast growth. **(B)** Effect of 3, 3.5, and 4 mM Gd on yeast growth. To find a sublethal concentration of Gd to *S. cerevisiae*, the effect of 0, 1, 2, 3, and 4 mM Gd on the yeast growth was observed, and 4 mM Gd was lethal for yeast. For 4 mM Gd was lethal for yeast, three additional Gd concentrations (3, 3.5, and 4 mM Gd) were further used to study the effect of Gd on yeast growth. The experiment was repeated three times.

We firstly tested the sensitivity of yeast cells to gadolinium nitrate in this study, and the experiment for screening Gd-sensitive phenotype of yeast was repeated three times. After cultivating yeast cells with gadolinium nitrate medium with different concentration gradients, it was observed that yeast cells had a specific sensitivity at 3.5 mM Gd (Figures 1A,B). Therefore, we confirmed screening the diploid mutant library of *S. cerevisiae* with 3.5 mM Gd (Figure 1B). Finally, we identified a total of 45 mutant sensitive to Gd and 10 resistance deletion mutant strains. In addition, the genotypes of mutants were further confirmed by PCR testing, and the result indicated that the genotypes of these mutants were correct (Supplementary Figures 1, 2).

**Figure 2:**
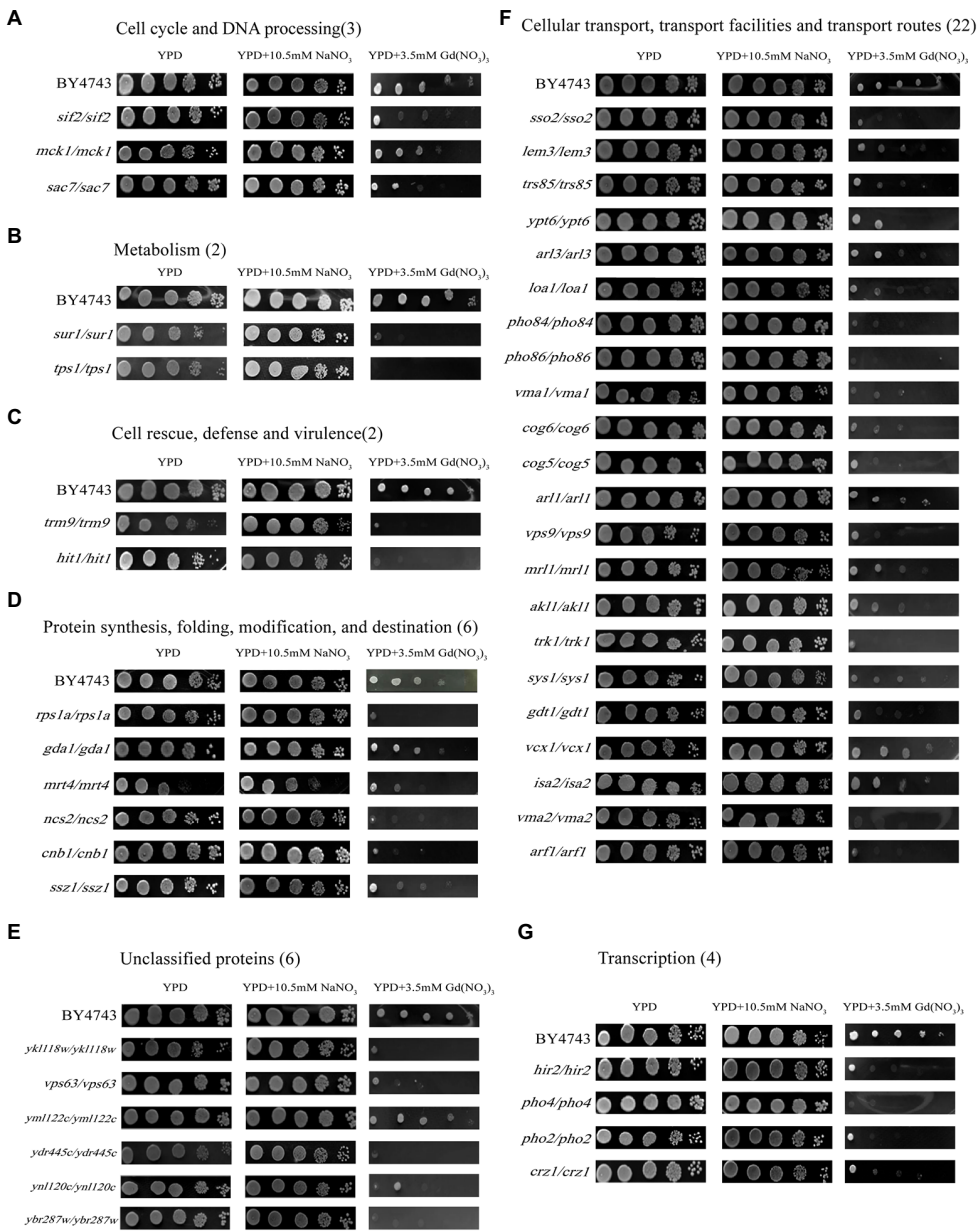
Phenotypes of Gd-sensitive gene deletion mutants. Five yeast culture diluents (10^−0^, 10^−1^, 10^−2^, 10^−3^, 10^−4^) were put on the YPD solid medium containing 0, 3.5 mM Gd(NO_3_)_3_, and 10.5 mM NaNO_3_, respectively. Cells of the wild-type BY4743 and 45 gene deletion mutants were identified from the genome-scale screen, and functional categories of the genes corresponding to the sensitive phenotypes were classified. **(A)** DNA processing and cell cycle (3 genes); **(B)** Metabolism (2 genes); **(C)** Cell rescue, virulence, and defense (2 genes); **(D)** Protein synthesis, modification, folding, and destination (6 genes); **(E)** Unclassified proteins (6 genes); **(F)** Cellular transport, transport routes, and transport facilities (22 genes); **(G)** Transcription (4 genes). The experiment was repeated three times.

Moreover, we performed the function and subcellular localization analysis of the sensitive genes by MIPS related network resources and SGD. By analyzing the functional classification, these Gd-sensitive genes are divided into the following 7 categories (Table 1; Figure 2; Supplementary Figure 3A). Group A, Metabolism (2 genes); Group B, DNA processing and cell cycle (3 genes); Group C, Transcription (4 genes); Group D, Protein synthesis, modification, folding, and destination (6 genes); Group E, Cellular transport, transport routes, and transport facilities (22 genes); Group F, Cell rescue, virulence, and defense (2 genes); Group G, Unclassified proteins (6 genes). Moreover, the sensitive genes are localized in cytoplasm (11 genes), Golgi bodies (10 genes), nuclei (9 genes), vesicles (7 genes), endoplasmic reticulum (4 genes), plasma membrane (4 genes), mitochondria (1 gene), and 5 unidentified location genes (Supplementary Figure 3A).

**Table 1:** Localization and function of genes related to Gd-sensitive deletion mutants.

Systemic name	Standard name	Gene function	Subcellular localization
**Metabolism (2)**			
YPL057C	*SUR1*	Mannosylinositol phosphorylceramide synthase catalytic subunit	Vacuole membrane
YBR126C	*TPS1*	Synthase subunit of trehalose-6-phosphate synthase/phosphatase complex	cytoplasm
**Cell cycle and DNA processing (3)**			
YBR103W	*SIF2*	WD40 repeat-containing subunit of Set3C histone deacetylase complex	nucleus
YNL307C	*MCK1*	Protein serine/threonine/tyrosine kinase involved in chromosome segregation and meiotic entry	nucleus
YDR389W	SAC7	Rho1p GTPase activating protein (GAP)	cytoplasm
**Transcription (4)**			
YOR038C	*HIR2*	Subunit of HIR nucleosome assembly complex involved in regulation of histone gene transcription	nucleus
YFR034C	*PHO4*	Basic helix–loop–helix transcription factor, regulatory targets include genes involved in phosphate starvation response (PHR)	nucleus
YDL106C	*PHO2*	Homeobox transcription factor, activates transcription cooperatively with Pho4p in response to phosphate starvation	nucleus
YNL027W	*CRZ1*	Transcription factor of the Ca^2+^ signaling pathway	nucleus
**Protein synthesis, folding, modification, and destination (6)**			
YLR441C	*RPS1A*	Ribosomal protein 10 of the small subunit	cytoplasm
YEL042W	*GDA1*	Guanosine diphosphatase in the Golgi lumen	Golgi
YKL009W	*MRT4*	Protein involved in mRNA turnover and ribosome assembly	nucleus (nucleolus)
YNL119W	*NCS2*	Protein involved in tRNA wobble position uridine thiolation	cytoplasm
YKL190W	*CNB1*	Regulatory subunit of calcineurin	cytoplasm
YHR064C	*SSZ1*	Heat shock protein 70	cytoplasm
**Cellular transport, transport facilities, and transport routes (22)**			
YML123C	*PHO84*	High-affinity inorganic phosphate transporter and low-affinity manganese transporter	plasma membrane
YJL117W	*PHO86*	Endoplasmic reticulum resident protein; required for ER exit of the high-affinity phosphate transporter Pho84p	ER
YDL128W	*VCX1*	Vacuolar membrane antiporter with Ca^2+^/H^+^ and K^+^/H^+^ exchange activity	vacuole membrane
YBR187W	*GDT1*	Ca^2+^ and Mn^2+^ transporter with higher affinity for Ca^2+^	Golgi apparatus/ vacuole membrane
YJL129C	*TRK1*	Component of the Trk1p-Trk2p potassium transport system	plasma membrane
YNL323W	*LEM3*	Membrane protein of the plasma membrane and ER involved in phospholipid translocation	plasma membrane/ ER
YMR183C	*SSO2*	Plasma membrane t-SNARE	Plasma membrane
YDL192W	*ARF1*	ADP-ribosylation factor	Golgi
YBR164C	*ARL1*	Soluble GTPase involved in regulation of membrane traffic	Golgi apparatus /cytosol
YPL051W	*ARL3*	ARF-like small GTPase	Golgi
YJL004C	*SYS1*	Integral membrane protein of the Golgi	Golgi membrane
YPR139C	*LOA1*	Lysophosphatidic acid acyltransferase	ER/lipid droplets
YDL185W	*VMA1*	Subunit A of the V1 peripheral membrane domain of V-ATPase	vacuole membrane
YBR127C	*VMA2*	Subunit B of V1 peripheral membrane domain of vacuolar H^+^-ATPase	Vacuolar membrane
YNL051W	*COG5*	Component of the conserved oligomeric Golgi complex	Golgi
YNL041C	*COG6*	Component of the conserved oligomeric Golgi complex	Golgi
YML097C	*VPS9*	Guanine nucleotide exchange factor and ubiquitin receptor;	cytoplasm
YPR079W	*MRL1*	Membrane protein	vacuole membrane
YBR059C	*AKL1*	Ser-Thr protein kinase;	cytoplasm
YLR262C	*YPT6*	Rab family GTPase	Golgi / cytosol
YDR108W	*TRS85*	Subunit of transport protein particle complex III	cytoplasmic vesicle/ Golgi apparatus
YPR067W	*ISA2*	Protein required for maturation of mitochondrial [4Fe-4S] proteins	mitochondrion
**Cell rescue, defense, and virulence (2)**			
YML014W	*TRM9*	tRNA methyltransferase	Cytoplasm / nucleus
YJR055W	*HIT1*	Protein involved in box C/D snoRNP assembly	nucleus
**Unclassified proteins (6)**			
YKL118W		unknown	unknown
YLR261C	*VPS63*	unknown	unknown
YML122C		unknown	unknown
YDR445C		unknown	unknown
YNL120C		unknown	unknown
YBR287W		unknown	ER

### Genes involved in the Gd resistance of yeast cells

**Figure 3:**
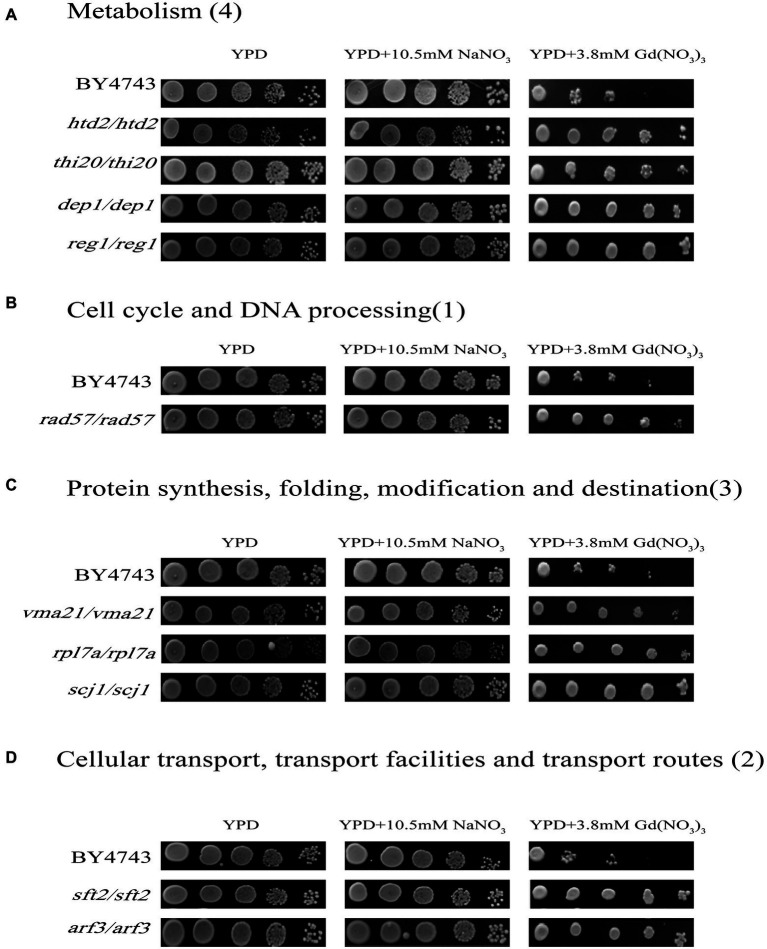
Phenotypes of Gd-resistant gene deletion mutants. Five yeast culture diluents (10^−0^, 10^−1^, 10^−2^, 10^−3^, 10^−4^) were put on the YPD solid medium containing 0, 3.8 mM Gd(NO_3_)_3_, and 10.5 mM NaNO_3_, respectively. Cells of the wild-type BY4743 and 10 gene deletion mutants were identified from the genome-scale screen, and functional categories of the genes corresponding to the resistant phenotypes were classified. **(A)** Metabolism (4 genes); **(B)** DNA processing and cell cycle (1 gene); **(C)** Protein synthesis, modification, folding, and destination (3 genes); **(D)** Cellular transport, transport routes, and transport facilities (2 genes). The experiment was repeated three times.

In this study, we confirmed a total of 10 Gd resistance deletion mutant strains (Table 2; Figure 3; Supplementary Figure 3B), and the experiment for screening Gd-resistant phenotype of yeast was repeated three times. In addition, the result of PCR testing indicated that the genotypes of these mutants were correct (Supplementary Figure 2). The Gd resistance genes were involved in 4 groups, which are as follows (Supplementary Figure 3B). Group H, Metabolism (4 genes); Group I, DNA processing and cell cycle (1 gene); Group J, Protein synthesis, modification, folding, and destination (3 genes); Group K, Cellular transport, transport routes, and transport facilities (2 genes). The Gd resistance genes are localized in the cytoplasm (4 genes), nuclei (2 genes), mitochondria (1 gene), endoplasmic reticulum (2 genes), and Golgi apparatus (1 gene).

**Table 2:** Localization and function of genes related to Gd-resistance deletion mutants.

Systemic name	Standard name	Gene function	Subcellular localization
**Metabolism (4)**			
YHR067W	*HTD2*	3-hydroxyacyl-thioester dehydratase in mitochondrion	mitochondrion
YOL055C	*THI20*	Trifunctional enzyme of thiamine biosynthesis, degradation and salvage	cytoplasm
YAL013W	*DEP1*	Component of the Rpd3L histone deacetylase complex	nucleus
YDR028C	*REG1*	Regulatory subunit of type 1 protein phosphatase Glc7p	cytoplasm
**Cell cycle and DNA processing(1)**			
YDR004W	*RAD57*	Protein that stimulates strand exchange	nucleus
**Protein synthesis, folding, modification and destination(3)**			
YGL076C	*RPL7A*	Ribosomal protein RPL7A of the large (60S) subunit	cytoplasm
YGR105W	*VMA21*	Protein involved in vacuolar H^+^-ATPase complex assembly	ER
YMR214W	*SCJ1*	One of chaperones involved in protein folding in the ER lumen	ER
**Cellular transport, transport facilities and transport routes (2)**			
YBL102W	*SFT2*	Tetra-spanning membrane protein found mostly in the late Golgi	Golgi
YOR094W	*ARF3*	Glucose-repressible ADP-ribosylation factor	cytoplasm

### The intracellular Gd content in the Gd sensitive and resistant mutants

**Figure 4:**
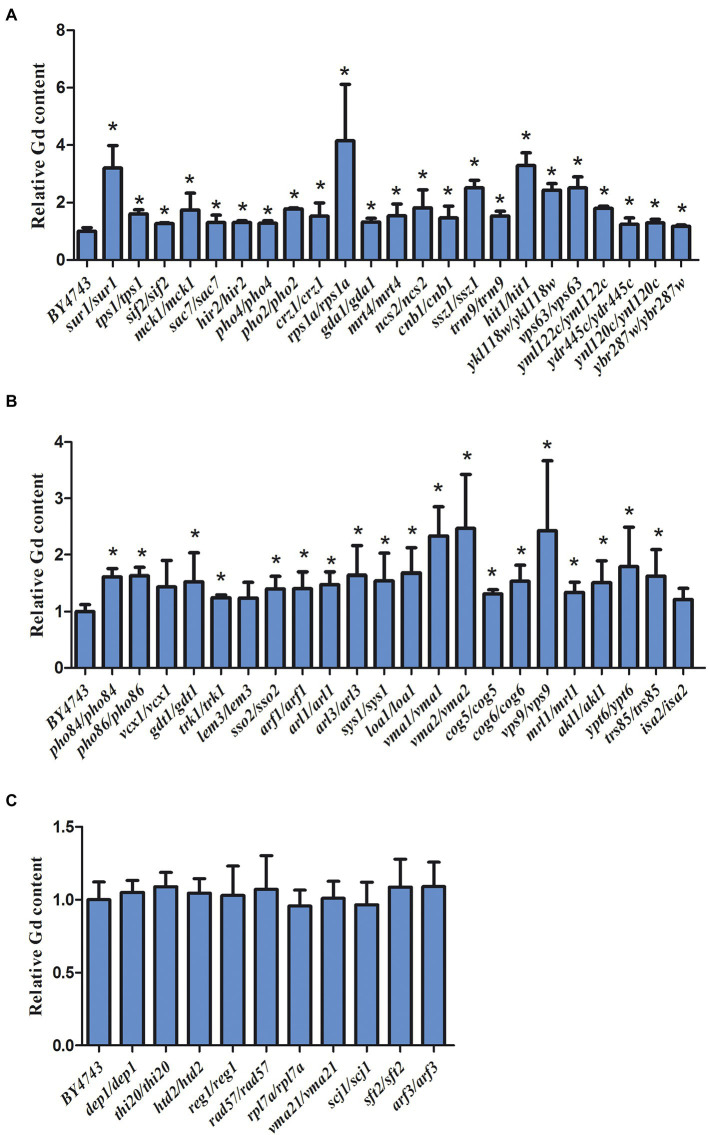
Intracellular Gd concentration in 45 Gd-sensitive and 15 Gd-resistant gene deletion mutants under Gd stress. **(A)** Intracellular Gd concentration in 23 Gd-sensitive gene deletion mutants; **(B)** Intracellular Gd concentration in 22 Gd-sensitive gene deletion mutants; **(C)** Intracellular Gd concentration in 15 Gd-resistant gene deletion mutants. Data was expressed as mean ± SEM (n = 6). The difference was analyzed with the single factor variance analysis (LSD’s test) analysis, and the level of significance is set at *p* < 0.05. Statistically significant differences are denoted by asterisk (*) (*p* < 0.05). The experiment was repeated six times.

The intracellular Gd concentration was detected and the experiment was repeated six times. Compared to the wild-type yeast, 42 sensitive deletion mutants had significantly higher intracellular Gd content than that in the wild-type cells except for 3 mutants *vcx1*, *lem3*, and *isa2* (Figure 4). The intracellular Gd content in 36 sensitive deletion mutants was about 1-fold of the wild-type cell (Figure 4). In addition, the intracellular Gd content in the mutants related to *ssz1*/*ssz1*, *ykl118w*/*ykl118w*, *vma1*/*vma1*, *vma2*/*vma2*, *vps9*/*vps9* Gd sensitive gene deletion mutants was about 2-fold of the wild-type cell (Figure 4A,B). The intracellular Gd content in the mutants related to *hit1*/*hit1* and *sur1*/*sur1* Gd sensitive gene deletion mutants were about 3-fold, and Gd content in the mutants *rps1a*/*rps1a* was about 4-fold of the wild-type cells (Figure 4A). Nevertheless, no remarkable difference on the intracellular Gd content was observed in 6 Gd resistance gene deletion mutants compared to wild-type BY4743 (Figure 4C).

### Correlation analysis between the functional categories of Gd sensitivity and resistance genes and proteins

**Figure 5:**
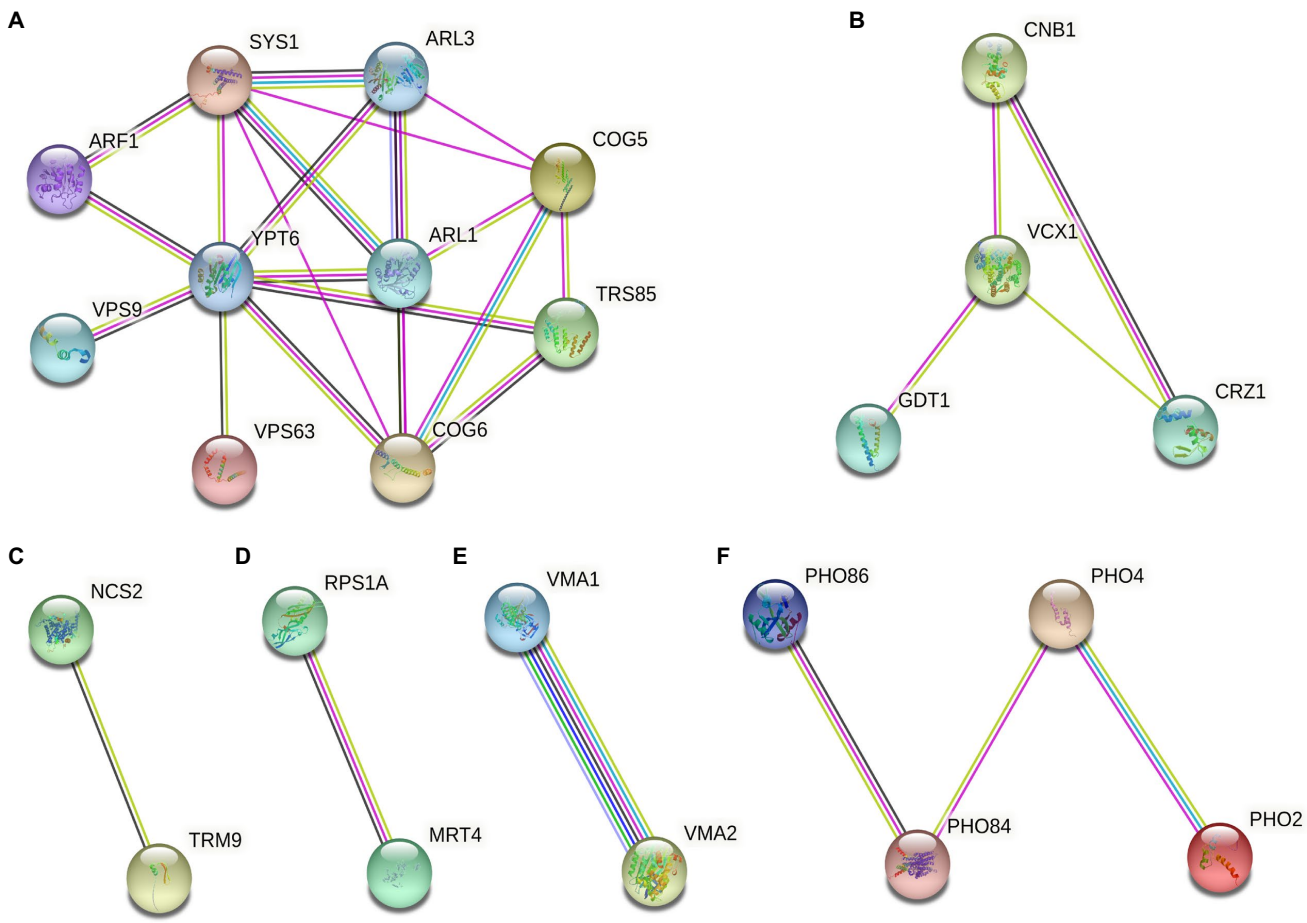
Gd toxicity modulating networks identified with proteins whose absence renders cells sensitive to Gd. **(A)** Gd sensitive genes (*ARF1*, *ARL1*, *ARL3*, *SYS1*, *COG5*, *COG6*, *YPT6*, *VPS9*, *SSO2*, *MRL1*, *AKL1*, and *TRS85*) were associated with the intracellular vesicle transport; **(B)** Gd sensitive genes (*CNB1, MCK1, VCX1, and CRZ1*) were involved in calcium signaling pathway; **(C)** Gd sensitive genes *NCS2* and *TRM9* were associated with the modification of tRNA wobble position; **(D)** Gd sensitive genes *PRS1A* and *MRT4* participated in protein synthesis; **(E)** Gd sensitive genes *VMA1* and *VMA2* were the subunit A and Subunit B of V1 peripheral membrane domain of V-ATPase; **(F)** Gd sensitive genes (*PHO84*, *PHO86*, *PHO2*, and *PHO4*) participated in phosphorus metabolism. The proteins corresponding to Gd sensitive gene deletion strains were mapped onto the interactome, and then filtered to identify the connected groups of proteins (https://cn.string-db.org/). The minimum required interaction score was set at high confidence 0.7. The straight lines indicate the protein–protein interactions.

For 55 deletion mutant strains were screened, we further performed protein function analysis on these genes (Figure 5). The result showed that these gene encode proteins were mainly related to intracellular vesicle transport, calcium signaling pathway, modification of tRNA wobble position, protein synthesis, composition of V-ATPase, and phosphorus metabolism (Figure 5). Notably, the most abundant of Gd sensitive genes (*ARF1*, *ARL1*, *ARL3*, *SYS1*, *COG5*, *COG6*, *YPT6*, *VPS9*, *SSO2*, *MRL1*, *AKL1*, and *TRS85*) were associated with the intracellular vesicle transport (Figure 5A). Some Gd sensitive genes (*CNB1, MCK1, VCX1, and CRZ1*) were involved in calcium signaling pathway (Figure 5B). Gd sensitive genes *NCS2* and *TRM9* were associated with the modification of tRNA wobble position (Figure 5C). In addition, Gd sensitive genes *PRS1A* and *MRT4* participated in protein synthesis (Figure 5D). Gd sensitive genes *VMA1* and *VMA2* were the subunit A and Subunit B of V1 peripheral membrane domain of V-ATPase (Figure 5E). Gd sensitive genes (*PHO84*, *PHO86*, *PHO2*, and *PHO4*) participated in phosphorus metabolism (Figures 5F).

## Discussion

In this study, the global effect and regulatory mechanism of Gd on yeast were investigated by genome-scale screening. We firstly tested the sensitivity of yeast cells to Gd by cultivating yeast cells in the different concentration of Gd. It is observed that yeast cells have a specific sensitivity at 3.5 mM Gd. In addition, we find that the yeast mutants lacking *ARF1*, *ARL1*, *ARL3*, *SYS1*, *COG5*, *COG6*, *YPT6*, *VPS9*, *SSO2*, *MRL1*, *AKL1*, and *TRS85* are significantly sensitive to Gd stress. These genes are involved in the process of vesicle trafficking. *ARL1* is a key coding gene of the Arf/Arl/Sar family, and *YPT6* is a key coding gene in the Rab family. Both of Arf/Arl/Sar family and Rab family play a key regulatory role in vesicle trafficking. In addition, *ARL1* and *YPT6* are present on the opposite membrane of Golgi network (TGN) and regulate vesicular trafficking between TGN and early endosomes (Rosenwald et al., 2002). Similar to the other GTP-binding proteins, the activities of *ARL1* and *YPT6* are positively related with GTP concentration. However, if GTP is hydrolyzed to GDP, they remain in the cytosol but are not active (Li and Warner, 1996). Ypt6p is a protein similar to human Rab6p (Yang and Rosenwald, 2016). *YPT6*, *ARF1*, and *ARL1* are important regulators of vesicle trafficking on the Golgi membrane. *TRS85* is a component of the transporter pellet (TRAPP) complex III. Moreover, TRAPP I and TRAPPIII, as the GEF of YPT1, can activate YPT1 through the subunit TRS85 and further participates in vesicle trafficking in the Golgi apparatus (Lynch-Day et al., 2010). *ARL3* requires the repositioning of Arl1p (a GTPase that regulates vesicular transport) to Golgi. *SYS1* is an integral membrane protein on Golgi, and is necessary for targeting Arf-like GTPase Arl3p to Golgi. *AKL1* is a Ser-Thr protein kinase, which belongs to the Ark kinase family (containing Ark1p and Prk1p) and participates in endocytosis and actin cytoskeleton formation (Roelants et al., 2017). The absence of the above 12 genes may affect intracellular vesicle trafficking, and Gd can not be effectively transported out of the cells.

In addition, *VPS9* participates in vacuolar protein sorting, and it is a transporter from the late Golgi complex to the storage vacuole precursor (PVC) (Bonangelino et al., 2002). In this study, the deletion of *VPS9* gene leads to the yeast cells being sensitive to Gd. However, we find that the intracellular Gd content in the *VPS9* yeast deletion strain is 2-fold higher than that in the wild-type yeast. Therefore, it indicates that the vacuolar septum also plays a key role in decreasing the intracellular Gd toxicity in yeast cells.

It is known that Ca^2+^ is one of the key signaling molecules and it is involved in regulating various cell activities (Berridge et al., 2003). Calcineurin (CaN), as a serine/threonine protein phosphatase, participates in various cellular metabolism processes and Ca^2+^-dependent signaling transduction pathways (Romano et al., 2017). In addition, *CNB1* is a gene encoding the CaN regulatory subunit (CnB) (Cunningham, 2011). The activity of CaN is regulated by *Rcn1*, whereas *Rcn1* activity is regulated by *MCK1*, a member of the GSK-3 kinase protein family (Hilioti et al., 2004). If yeast is exposed to the external environmental stress, the Ca^2+^/CaN signaling pathway will be activated. In the normal growing environment, there is adequate Ca^2+^ concentration in the cytoplasm and CaN can be activated. Thus, cells lacking *CNB1*, *MCK1*, *VCX1*, and *CRZ1* gene can grow normally in YPD medium. For the radius of Gd^3+^ is similar to Ca^2+^, Gd^3+^ can compete for binding with the site of Ca^2+^, which will reduce the intracellular Ca^2+^ levels and affect the Ca^2+^-dependent signaling transduction pathway. However, in a certain range of Ca^2+^ levels, it does not decrease yeast cell viability.

Moreover, CaN dephosphorylates the transcription factor Crz1 in the cytoplasm, and Crz1 will rapidly transfer from cytoplasm to nucleus (Bodvard et al., 2013). Crz1 further induces the target genes, including the calcium pump genes *PMC1* in the vacuolar membrane and *PMR1* in the membrane of ER and Golgi (Xu et al., 2019; Yan et al., 2020). These two calcium pumps (*PMC1* and *PMR1*) and the Ca^2+^/H^+^ exchange protein Vcx1 on the vacuole membrane cooperate to control Ca^2+^ concentration within the normal physiological concentration range in the cytoplasm (Miseta et al., 1999), which allows the cells to grow normally. However, the yeast cells lacking *CRZ1* and *VCX1* genes are difficult to survive under the Gd stress environment. In addition, Gdt1 is a Ca^2+^/H^+^ exchanger on the membrane of vacuole and Golgi, which is involved in regulating Ca^2+^ transmembrane transport. Gdt1 also plays a key role in maintaining the dynamic intracellular Ca^2+^ balance (Colinet et al., 2017; Dulary et al., 2018; Thines et al., 2020).

Gd sensitive genes *NCS2* and *TRM9* are associated with the modification of tRNA wobble position. Protein NCS2 is required for uridine thiolation at the wobble position of tRNA and plays function in protein urmylation (Noma et al., 2009). And, NCS2 has a role in regulating urmylation, invasive, and pseudohyphal growth. Protein TRM9 is a tRNA methyltransferase and it catalyzes modification of wobble bases in tRNA anticodons (Patil et al., 2012). It is involved in avoiding the deletion mutation in the translational infidelity, including amino acid misincorporation and frameshifting. In addition, Gd sensitive genes *PRS1A* and *MRT4* participated in protein synthesis (Zuk et al., 1999). In our study, Gd sensitive genes *NCS2*, *TRM9*, *PRS1A* and *MRT4* were identified in yeast. It shows that the yeast mutants of *NCS2*, *TRM9*, *PRS1A* and *MRT4* display the activation of Gd stress responses. The stress of Gd may affect the modification of tRNA wobble position and protein synthesis in yeast.

The H^+^-ATPase on the vacuolar membrane is composed of two complexes, including V_1_ and V_0_. V_1_ consists of eight subunits, which are responsible for ATP hydrolysis (Jefferies et al., 2008). Moreover, V_0_ consists of six subunits responsible for H^+^ transport. The absence of genes *VMA2* and *VMA1*, which encode the A and B subunits of V_1_ complex, makes the yeast cells sensitive to Gd. In addition, the intracellular Gd content was also significantly higher in the *VMA2* and *VMA1* gene deletion strains than that in the control.

Phosphorus is an essential and massive element for cell growth. Maintaining the dynamic balance of intracellular phosphorus is essential for cell survival. Once the phosphorus levels are unbalanced, it will affect cell differentiation and proliferation, disrupt cell metabolism, and seriously deform cytoskeletal morphology (Dick et al., 2011). In response to the dynamic phosphate levels in environment, yeast has evolved a signaling pathway (PHO pathway) to real-time monitor the intracellular phosphorus metabolism. In this study, we observed that the cells lacking *PHO84*, *PHO86*, *PHO2*, and *PHO4* genes were abnormally sensitive to Gd stress. The result is similar to the effect of reduced intracellular phosphorus levels or the formation of inactive phosphate precipitation (Mouillon and Persson, 2006; Wykoff et al., 2007; Dick et al., 2011). Moreover, in the five unidentified location genes, the *yml122c* mutant does not have a death ratio as much as the other 5 mutants. Five genes are highly sensitive to the Cd exposure while *yml122c* is resistant, which indicates this gene may have a crucial role in cell death mechanisms. Previously, the result shows that *yml122c* is also one member of the high-affinity PHO pathway (Haas, 2012). Thus, *yml122c* also plays a role in regulating phosphate levels. In addition, our result shows that four deletion strains also had higher intracellular Gd content than the wild-type yeast. Previously, we found that Gd and phosphate salts form an insoluble inactive state in rice seedlings (Zhang et al., 2019a). Therefore, Gd and phosphate salts may form an insoluble inactive state in yeast, and the intracellular absence of available phosphorus finally results in the cellular phosphorus deficiency stress.

In addition, LEM3 is a membrane protein of the plasma membrane and ER involved in phospholipid translocation. ISA2 is a protein required for maturation of mitochondrial [4Fe-4S] proteins. HIT1 is a protein involved in box C/D snoRNP assembly, and LOA1 isa lysophosphatidic acid acyltransferase. TRK1 is a component of the Trk1p-Trk2p potassium transport system, and GDA1 is a guanosine diphosphatase in the Golgi lumen. HIR2 is a subunit of HIR nucleosome assembly complex involved in regulation of histone gene transcription. SAC7 is a Rho1p GTPase activating protein (GAP), and SIF2 is a WD40 repeat-containing subunit of Set3C histone deacetylase complex. Moreover, TPS1 is a synthase subunit of trehalose-6-phosphate synthase/phosphatase complex. However, the detailed function of these proteins needs to be further studied in future for there are less information on these proteins.

## Conclusion

In summary, the global effect and regulatory mechanism of Gd on yeast were investigated by genome-scale screening. Our result shows that 45 gene deletion strains are sensitive to Gd and 10 gene deletion strains are Gd-resistant from the diploid gene deletion strain library of *S. cerevisiae*. The localization analysis shows that most of these genes are involved in cell metabolism, cell cycle, transcription, translation, protein synthesis and folding, cell transport, etc. The result of functional analysis shows that four genes (*CNB1*, *CRZ1*, *VCX1*, and *GDT1*) are involved in the calcium signaling pathway, and four genes (*PHO84*, *PHO86*, *PHO2*, and *PHO4*) are involved in phosphorus metabolism. For Gd^3+^ has the similar ion radius with Ca^2+^ and easily binds to the phosphate radical, it further affects the Ca^2+^ signaling pathway and phosphorus metabolism. The genes *ARF1*, *ARL1*, *ARL3*, *SYS1*, *COG5*, *COG6*, *YPT6*, *VPS9*, *SSO2*, *MRL1*, *AKL1*, and *TRS85* participate in protein sorting and vesicle transport. In addition, the intracellular Gd content in the 45 sensitive deletion strains is higher than that in the wild type yeast under Gd stress. It suggests that the sensitivity of yeast deletion strains may be related to the excessive intracellular Gd accumulation.

## Data availability statement

The original contributions presented in the study are included in the article/Supplementary material, further inquiries can be directed to the corresponding author.

## Author contributions

XW: conceptualization, supervision. YL, KL, and JD: methodology. YC and CZ: writing–original draft preparation. YC, CZ, and YF: investigation. YC and XW: writing–reviewing and editing. All authors contributed to the article and approved the submitted version.

## Funding

This study was supported by National Natural Science Foundation of China (30900071).

## Conflict of interest

The authors declare that the research was conducted in the absence of any commercial or financial relationships that could be construed as a potential conflict of interest.

## Publisher’s note

All claims expressed in this article are solely those of the authors and do not necessarily represent those of their affiliated organizations, or those of the publisher, the editors and the reviewers. Any product that may be evaluated in this article, or claim that may be made by its manufacturer, is not guaranteed or endorsed by the publisher.

## Footnotes

^1^ http://www.yeastgenome.org

^2^ http://funspec.med.utoronto.ca

^3^ http://www.thebiogrid.org

^4^ https://cn.string-db.org/
