# Supplementary material for: Investigation the global effect of rare earth gadolinium on the budding Saccharomyces cerevisiae by genome-scale screening
Source: Front Microbiol. 2022 Nov 28;13:1022054. doi: 10.3389/fmicb.2022.1022054 (PMC9742279; doi:10.3389/fmicb.2022.1022054)
Supplement: Supplementary file 1 [file Image_1.pdf]

Supplementary Figure 1

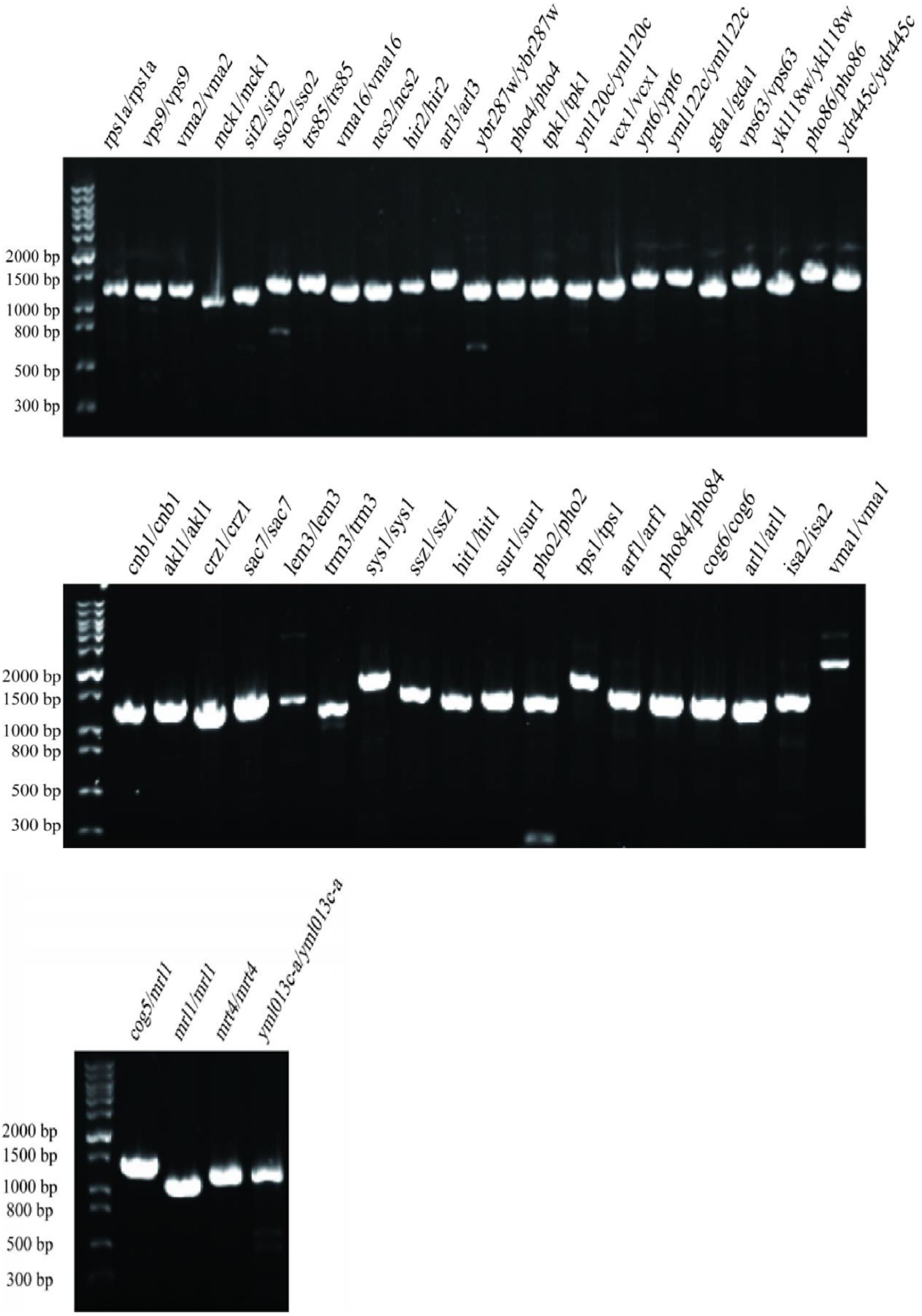

Supplementary Fig 1. Gd-sensitive deletion strain genotype testing by PCR.
