# Supplementary material for: Investigation the global effect of rare earth gadolinium on the budding Saccharomyces cerevisiae by genome-scale screening
Source: Front Microbiol. 2022 Nov 28;13:1022054. doi: 10.3389/fmicb.2022.1022054 (PMC9742279; doi:10.3389/fmicb.2022.1022054)
Supplement: Supplementary file 2 [file Image_2.pdf]

Supplementary Figure 2

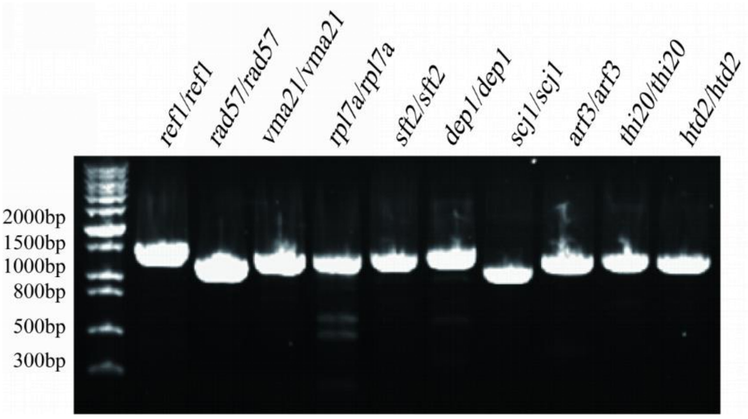

Supplementary Fig 2. Gd-resistance deletion strain genotype testing by PCR.
