# Supplementary material for: Investigation the global effect of rare earth gadolinium on the budding Saccharomyces cerevisiae by genome-scale screening
Source: Front Microbiol. 2022 Nov 28;13:1022054. doi: 10.3389/fmicb.2022.1022054 (PMC9742279; doi:10.3389/fmicb.2022.1022054)
Supplement: Supplementary file 3 [file Image_3.pdf]

Supplementary Figure 3

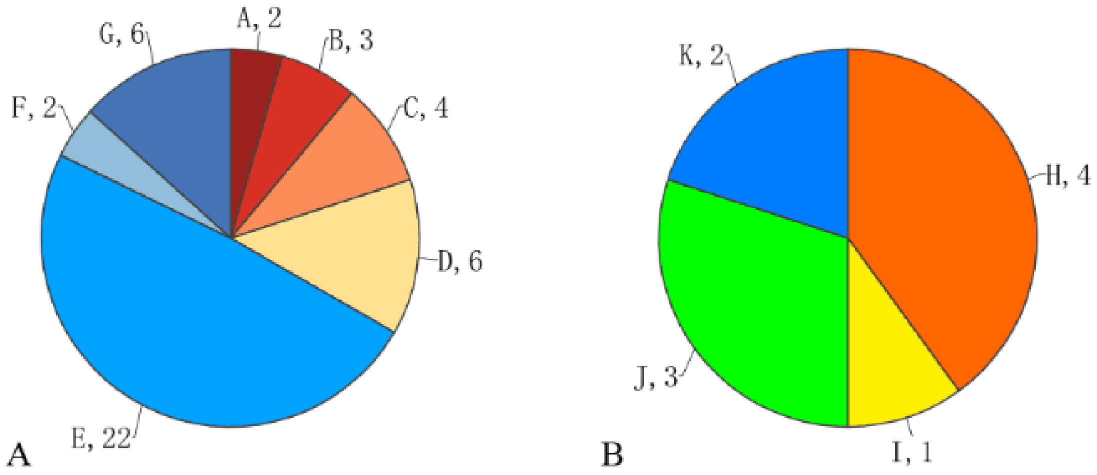

Supplementary Fig 3. Functional categories of the genes corresponding to the sensitive and resistant phenotypes and number of genes associated with each category. (A) Functional categories of the genes corresponding to the sensitive phenotypes. Group A, Metabolism (2 genes); Group B, DNA processing and cell cycle (3 genes); Group C, Transcription (4 genes); Group D, Protein synthesis, modification, folding, and destination (6 genes); Group E, Cellular transport, transport routes, and transport facilities (22 genes); Group F, Cell rescue, virulence, and defense (2 genes); Group G, Unclassified proteins (6 genes). (B) Functional categories of the genes corresponding to the resistant phenotypes. Group H, Metabolism (4 genes); Group J, Protein synthesis, modification, folding, and destination (3 genes); Group K, Cellular transport, transport routes, and transport facilities (2 genes); Group I, DNA processing and cell cycle (1 gene).
