# Supplementary material for: Investigation the global effect of rare earth gadolinium on the budding Saccharomyces cerevisiae by genome-scale screening
Source: Front Microbiol. 2022 Nov 28;13:1022054. doi: 10.3389/fmicb.2022.1022054 (PMC9742279; doi:10.3389/fmicb.2022.1022054)
Supplement: Supplementary file 4 [file Table_1.docx]

**Supplementary table 1**

**Table 1.** PCR Primers used in this study

| **Primer name** | **Sequence (5′~3′)** |
| --- | --- |
| KanMX4-F | gataatgtcgggcaatcagg |
| REG1-R | GACTGTTGTGTGGTGTCTTC |
| VMA21-R | GGATATCACATATGGTGCGTTG |
| VPS9-R | GTGCTTAGGAAGGTACGTG |
| RPS1A-R | GTCACACAGAGCAGAAATACC |
| THI20-R | CAAATGGTTCGTGATGAGGG |
| VMA1-R | GGGGTGTAAACCATTGCC |
| COG6-R | GAAAGCCCCGTAAGAACC |
| COG5-R | GGACAGAATGACCACGTA |
| ARL1-R | GGCCTTCTCATTCATCGG |
| PHO84-R | ACGTTTCGTGGTTTCCAG |
| MRL1-R | TATGGTGTGTGGGGGATCA |
| RAD57-R | GCGAGGATTGAATAATGTGC |
| RPL7A-R | TAGAGAAGTCAAGGCCGC |
| ARF1-R | ATGATTCACGCAGGGGTG |
| SFT2-R | CTCATGGTAACGAGCCTCC |
| SSO2-R | GGACTAATATTAAGGGCGACAG |
| LEM3-R | TAACCGGTACTGTAGGCG |
| TRS85-R | GAGCTAACGGGTAATGCAAG |
| SAC7-R | CAAGCTGTAACATTTCTCTGGC |
| GDA1-R | GGATAAACCTCGGAAGGCC |
| MRT4-R | GCTCTCACGTTCTTCCTTATTG |
| YKL118W-R | GGCTAGTATTCGTCTTCCTAGA |
| CNB1-R | GAGATCAGCTTCCATCTTGG |
| VPS63-R | CGTTACTGAGAAATGGCCG |
| YPT6-R | GCCGTTATGTGGACACAG |
| LOA1-R | GCCGAGATAAGGTGGAGA |
| PHO4-R | CCGTGTGTGTAGGTGTAAG |
| PHO86-R | CTCTTCAAGGTGTACTCTGC |
| ISA2-R | GAGATGGAAGGAAGTTCTCGA |
| VMA2-R | CAGGGTGATGTGTTCCTTG |
| TRM9-R | TCTTCTGTGTGCGAAAGG |
| SYS1-R | AGAGGTGTTCAGGACTGG |
| CRZ1-R | AGAAAATTCAGACGGGCG |
| VCX1-R | AATAGGGCTTCGCGTCTC |
| MCK1-R | TCCGAGGGGAAAGAGAAC |
| GDT1-R | CTTCATTGGGTTGGCTGG |
| SSZ1-R | CTGAATGGCACGATAAGCC |
| HIT1-R | CAAGTCTGCTTGACGCCT |
| SUR1-R | GTATGTCGTAAGAAGCGAGC |
| PHO2-R | CACCTATAACGCGAGCTTG |
| TPS1-R | CTACAGACAGGCGTTAACG |
| YDR445C-R | GACAGCTGCCATACCAGA |
| ARL3-R | GAGCAGATACGTCAATGTAGC |
| YML122C-R | CGACTCGGTATACTCTGCC |
| AKL1-R | CCACCTTTGTTCTTCCGG |
| SIF2-R | TCGAGATTCCTGCACAAC |
| DEP1-R | AATTCAATCTGGCACGGC |
| SCJ1-R | ATGCCTATTACCGGATGC |
| HTD2-R | AGGGAAGTACTTTGCGTC |
| NCS2-R | CAACTTCGACGTCCGTTC |
| YNL120C-R | GAAGTTGCCTGCACAGTG |
| TRK1-R | CGTTGACGATGACGAAAGC |
| YBR287W-R | CAAAGCTGTACAAGGGTGC |
| HIR2-R | CCTGTGTTGGCATTGGTATG |
| ARF3-R | CGGTAGTGTTGAAGTTACCG |
